# Community-based infant hearing screening in a developing country: parental uptake of follow-up services

**DOI:** 10.1186/1471-2458-9-66

**Published:** 2009-02-23

**Authors:** Bolajoko O Olusanya, Oladele O Akinyemi

**Affiliations:** 1 Institute of Child Health and Great Ormond Street Hospital for Children NHS Trust, University College London, 30 Guilford Street, London, WC1N 1EH, UK; 2 Maternal and Child Health Unit, Department of Community Health and Primary Care, College of Medicine, University of Lagos, Surulere, Nigeria; 3 Nigerian Dyslexia Association, 286A Corporation Drive, Dolphin Estate, Ikoyi Lagos, Nigeria

## Abstract

**Background:**

Universal newborn hearing screening is now considered an essential public health care for the early detection of disabling life-long childhood hearing impairment globally. However, like any health interventions in early childhood, parental support and participation is essential for achieving satisfactory uptake of services. This study set out to determine maternal/infant socio-demographic factors associated with follow-up compliance in community-based infant hearing screening programmes in a developing country.

**Methods:**

After health educational/counselling sessions, infants attending routine childhood immunisation clinics at four primary care centres were enrolled into a two-stage infant hearing screening programme consisting of a first-stage screening with transient-evoked otoacoustic emissions and second-stage screening with automated auditory brainstem response. Infants referred after the second-stage screening were scheduled for diagnostic evaluation within three months. Maternal and infant factors associated with completion of the hearing screening protocol were determined with multivariable logistic regression analysis.

**Results:**

No mother declined participation during the study period. A total of 285 out of 2,003 eligible infants were referred after the first-stage screening out of which 148 (51.9%) did not return for the second-stage, while 32 (39.0%) of the 82 infants scheduled for diagnostic evaluation defaulted. Mothers who delivered outside hospitals were significantly more likely to return for follow-up screening than those who delivered in hospitals (Odds ratio: 1.62; 95% confidence intervals: 0.98 – 2.70; p = 0.062). No other factors correlated with follow-up compliance for screening and diagnostic services.

**Conclusion:**

Place of delivery was the only factor that correlated albeit marginally with infant hearing screening compliance in this population. The likely influence of issues such as the number of return visits for follow-up services, ineffective tracking system and the prevailing unfavourable cultural perception towards childhood deafness on non-compliance independently or through these factors warrant further investigation.

## Background

Early detection of infants with permanent congenital or early-onset hearing loss (PCEHL) is essential for optimal development of affected children in early childhood and this has resulted in an increasing implementation of universal infant hearing screening programmes worldwide [1-5]. Such programmes require mothers to make informed choice about enrolling their children for the initial screening and for subsequent stages right up to diagnosis for those who failed the screening tests. While the possible effects of newborn hearing screening on maternal anxiety and parent-infant bonding have been debated [6,7], several studies in developed and developing countries have documented favourable views among majority of mothers on the need for early detection of PCEHL [4,8-10]. For instance, reported participation in the first-stage screening under these programmes is often impressive with uptake as high as 98% [5,11]. However, a major challenge in most countries is how to ensure that all parents of babies who fail the initial screening test and are at risk of PCEHL return for subsequent follow-up appointments.

The Joint Committee on Infant Hearing Screening (JCIH) of USA for instance recommends outpatient re-screening of infants referred in the first-stage screening by 1 month of age with a view to achieving a minimum coverage of 95% [2]. The effectiveness of screening programmes is compromised when timely detection of PCEHL is forestalled by failure to complete the process. At the population level, reliably estimating the incidence of this condition becomes difficult and this has implications for the planning and development of appropriate intervention services.

The problem of non-compliance is perhaps more prominent in developing countries where facilities for effective tracking of mothers are lacking. For instance, in a recently concluded community-based infant hearing screening pilot programme in Lagos, Nigeria over half (52%) of mothers who were required to present their children for further evaluation after failing the initial screening test did not return regardless of the incentives of free transportation and no fee for all services up to the provision of amplification devices if required [12]. Similar rates of default have been reported in other developing countries [5].

Socio-demographic characteristics such as maternal education, ethnicity and parity; economic status as well as infant medical history such as prematurity and hospital admission in the neonatal period have been associated with follow-up default in infant hearing screening programmes [11,13,14], similar to findings in other screening programmes [15,16]. For instance, one study found that socio-demographic factors such as young maternal age, having more than two children at home (parity), being non-white (race), substance abuse, late onset of prenatal care and lack of health insurance were predictive of non-compliance with hearing screening protocol [13]. Another study reported that infants with characteristics such as low birth weight, being white (race) and born to women who had not completed high school were almost twice as likely not to complete newborn hearing screening compared to non-white infants or those with normal birth weight [14]. However, informed parental education has been found to be effective in modifying health-seeking behaviour over a range of health interventions in the developing world despite the challenges of low education and literacy levels [17].

This study therefore set out to compare the characteristics of mothers who did not complete the screening process with those who completed to identify factors that may help to improve pre-screening parental education and counselling towards minimising loss to follow-up in community-based infant hearing screening programmes in a developing country.

## Methods

### Design

Cross-sectional community-based study conducted in an inner-city area of Lagos, Nigeria with a population of 243,777. Ethical approvals for this study were obtained from University College London, UK and Lagos State Health Management Board, Nigeria as part of a wider doctoral work by BOO [12] and in line with the Helsinki Declaration.

### Participants

The participants in this study were mothers of all infants who failed a hearing screening test and who were scheduled for additional tests one week after the initial screening. They were drawn from a population of mothers previously described [12] who were enrolled for a three-stage infant hearing screening programme at the time of attending four community health centres to obtain Bacille de Calmette-Guérin (BCG) vaccinations for their babies. The BCG vaccination was administered once a week at each centre and the average attendance was 15. All the four centres were located within a radius of about 2 miles well served by public transportation. Moreover, under Nigeria's health policy primary healthcare centres are located close to the population being served to ensure high uptake of services. However, free transportation was provided under our programme principally to convey mothers and their babies comfortably from the screening sites to the diagnostic centre even though the centre was also readily accessible by public transportation.

### Pre-screening education for community health workers

**Figure 1:**
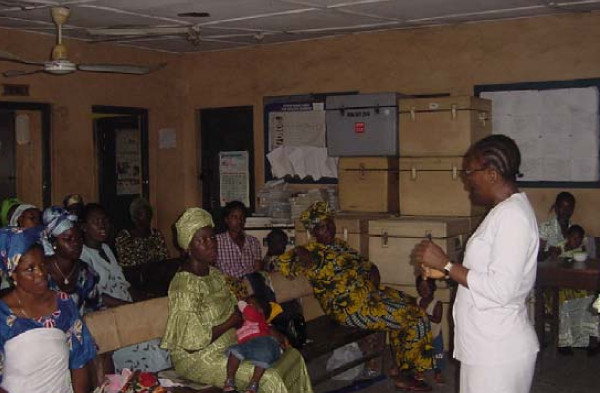
**A typical session on parental education at a community health centre**.

An awareness workshop on the significance and purpose of the screening project was briefly conducted by the principal investigator for the community nurses at each centre a week before the commencement of screening programme, during which the screening team was also introduced. The community nurses were charged with the responsibility of educating parents about the screening programme during the routine pre-vaccination health talks because of their vast experience in communicating with mothers while members of the screening team were also in attendance (Figure 1). The workshop addressed consequences of PCEHL, benefits of early detection, nature of the screening tests and follow-up services that will be provided for children detected with PCEHL. The possibility of false-negatives and false-positives with screening tests was also highlighted during parental education to emphasise the need for completing the entire screening protocol and the importance of on-going surveillance. Post-training evaluation showed that the community nurses were pleased with the programme objectives particularly with the fact that the screening tests were quick, painless and non-invasive. They were also pleased to learn that all the required services under the programme will be provided free including transportation to the diagnostic centre for those who required this evaluation and the provision of hearing aids. As part of the educational programme an information booklet addressing some important issues relevant to infant hearing screening programme in Nigeria (Table 1) was distributed to all the health workers with additional copies made available for all parents participating in the programme. Both written and verbal information addressing the benefits and limitations of the screening programme were provided for parents before their consent was sought to enrol their children.

**Table 1:** Educational booklet for parents and primary healthcare workers on infant hearing screening programme in Nigeria

	**Topical questions**
*1*.	*What happens when a child is unable to hear?*
*2*.	*Is hearing loss a common problem in Nigerian children?*
*3*.	*What are the main causes of childhood hearing loss?*
*4*.	*Are the risk factors for hearing loss preventable?*
*5*.	*What can be done when primary prevention fails?*
*6*.	*What does the screening test entail?*
*7*.	*What happens when the screening results are unsatisfactory?*
*8*.	*How can the government help parents?*
*9*.	*What role can the public play to support government?*
*10*.	*What is the healthcare worker's role?*

A booklet addressing these 10 questions can be found at the following website:

### Pre-screening parental education

Prior to screening each day, the trained community nurses educated parents on the purpose and benefits of the screening programme with the aid of a patient information leaflet specially produced for the programme and in line with the training they had received. The patient leaflet contained simple-worded milestones for speech and language development from birth to age 3 years as well as a detachable section for recording parental consent. The screening programme was presented as part of the immediate post-delivery examination necessary to ensure that the newborns had no detectable hearing abnormality that could later impair normal speech and language development. The importance of follow-up appointments following a referral at any stage was emphasised. Parents were also informed that all the services to be provided under the programme including the provision of hearing aids were at no charge. Members of the screening team were always present to answer any questions or corroborate points already made by the community nurses. Thereafter, the parents were required to complete the consent form with the assistance of a member of the screening team who also elicited the medical history and demographic details from the mother. In line with studies examining the socio-demographic profile of participants in relation to health outcomes or behaviours [11,13,18], the variables elicited were maternal age (subsequently categorised into: below 20 years, 20 to 35 years and above 35 years), ethnicity (based on the three most predominant tribes in Nigeria: Hausa, Ibo and Yoruba); marital status, parity (later grouped into primiparous and multiparous) and religion (Christianity and Islam). Maternal and spouses' education and occupation were also obtained. Four educational levels were used: none (zero years of formal schooling), primary (1 – 6 years of formal schooling), secondary (7 – 12 years of formal schooling) and tertiary (more than 12 years of formal schooling including university education). Occupation was grouped into four: none (for unemployed or full-time house wife), small trade (like petty trading of merchandise or pepper grinding), casual labour (for contract and irregular employment) and full-time employment (reflecting regular and predictable stream of income). Infants characteristics studied included gender, age at screening, gestational age (<37 weeks and 37 weeks or over), place of delivery grouped into hospital (private and public) and non-hospital (traditional maternity homes, family homes, church premises and born before arrival at a birthing facility). Hospital admission in the first 28 days of life was included as an index of any serious illness that could not be determined reliably from mothers especially among those who delivered outside hospitals.

### Screening procedure

Details of the screening programme have been described elsewhere [12]. In summary, a two-stage screening protocol was implemented consisting of an initial screening with transient evoked otoacoustic emissions (TEOAE) followed by a second-stage screening with automated auditory brainstem response (AABR) for all first-stage referrals. Both instruments were fully automated to display a "pass" or "refer" test outcome. "Refer" outcomes during the various stages of screening in particular were not presented as evidence of hearing loss but rather as indication that further tests were required to rule out any uncertainty regarding the hearing status of the child. As much as possible screening was performed before BCG vaccination but in exceptional cases or for logistic reasons it followed vaccination. Babies who were referred by this initial screen were scheduled for re-screening within one week with AABR at one of the four community centres designated for this purpose. Those who failed AABR screening were scheduled for diagnostic evaluation which was the third stage of the programme. Diagnostic evaluation was often conducted every Friday at an audiological centre within easy reach from all the four screening centres and consisted of tympanometry including high frequency (1000 Hz) probe tone for babies less than 4 months old, diagnostic tone pip ABR with insert ear phones and/or free-field visual reinforcement audiometry for babies older than 6 months. Mothers of babies who required re-screening or diagnostic evaluation were usually given the option of going to the designated centre directly or returning to the centre where the initial screening was conducted to be conveyed by a member of the screening staff to the appropriate location. Follow-up counselling appointments and intervention services were scheduled for parents of babies who were confirmed with any degree of bilateral or unilateral sensorineural hearing loss including provision of hearing aids where appropriate.

### Analysis

A data tracking and management software – HI*TRACK for Windows Version 3.5 Desktop (National Centre for Hearing Assessment and Management: NCHAM, Logan, UT, USA) – was used for monitoring the babies through the various stages of screening, referral and confirmatory procedures. Mothers who returned for and completed the second-stage screening or diagnostic evaluation were compared to those who did not return across the selected variables. Differences between groups were explored with two-tailed Pearson chi-square test or Fisher's exact test as appropriate (for variables with 5 or less records) to determine the odds ratio (OR) at 95% significance level. In addition, Student's t-test was used to compare the means of continuous variables (maternal age, infants' gestational age and age at screening). Factors predictive of hearing test completion were explored with multivariable logistic regression based on factors found to be significant (p < 0.10) in the univariable analyses. Possible interactions between variables entered into the regression model were evaluated with likelihood ratio test while goodness-of-fit of model was assessed with Hosmer-Lemeshow test. Model discriminatory powers were assessed by the c-statistic (indexed by the area under the receiver operating characteristic [ROC] curve and Nagelkerke *R*^2 ^statistic (a measure of explained variation in the model). SPSS Windows version 16.0 (SPSS Inc, Chicago, IL, USA) was used for all statistical analyses.

## Results

All the eligible 2,003 infants (male: 50.2% and female: 49.8%) were screened over the study period at an average chronological age of 17.1 (standard deviation: 19.1) days. About 84% of the infants were presented for BCG immunisation within the first month of life. All except 6 (0.3%) babies were singletons while only 15 (0.7%) were born preterm (less than 37 weeks). The mothers were predominantly (85.9%) in the age group of 20 – 35 years with a mean age of 28.0 years. About 3.2% (105) were teenage mothers, 2.3% (93) were unmarried and almost half (48.4%) were first-time mothers. Only a small percentage (1.6%) had no formal education while the vast majority (71.9%) were either engaged in petty trading (59.7%) or were unemployed (12.2%). Less than half (44.9%) delivered their babies in hospitals (data not shown).

**Figure 2:**
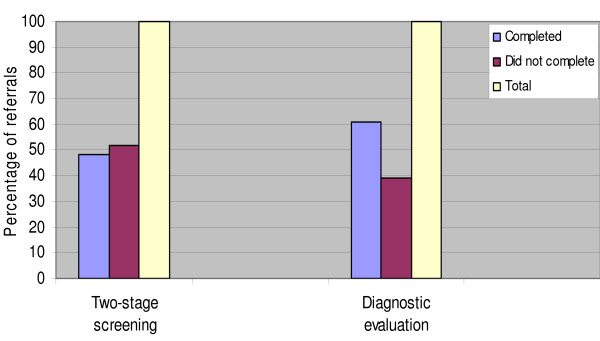
**Participation rates in an infant hearing screening programme in Nigeria**.

A total of 287 (14.3%) infants, all singletons, were referred at the first stage screening out of which two infants who were abandoned by their mothers and brought to the clinics by social workers were excluded from our analysis because of lack of information on their mothers. Of the remaining infants, less than half (48.1%: 137/285) returned for the second-stage (AABR) screening out of which 59.9% (82/137) were referred for diagnostic evaluation (Figure 2). A total of 50 (61.0%) infants and their mothers returned for diagnostic evaluation and 45 (90.0%) infants were confirmed with hearing loss. In effect, 148 (51.9%) mothers scheduled for second-stage screening defaulted and 32 (39.0%) of those scheduled for diagnostic evaluation did not return.

As shown in Table 2, majority of mothers included in this study were married; between ages 20 and 35; belonged to Yoruba ethnic group; were multiparous; shared the Islamic faith; and had a minimum of secondary education. About 60% were engaged in small trade but majority of their spouses were in full-time employment. Majority of infants were full-term at birth and were predominantly delivered outside hospitals (Table 3). They were mostly one month old at first-stage screening and only a few were admitted in hospital for an illness within the first month of life.

**Table 2:** Socio-demographic profile of mothers who completed or did not complete the infant hearing screening programme

**Profile**	**Two-stage screening**		**Diagnosis**	
	
	**Did not complete (%)** n = 148	**Completed (%)** n = 137	**Did not complete (%)** n = 32	**Completed (%)** n = 50
Age (Years)				
<20	3 (2.0)	2 (1.5)	1 (3.1)	1 (2.0)
20 – 35	129 (87.2)	117 (86.7)	30 (93.8)	41 (82.0)
>35	16 (10.8)	16 (11.9)	1 (3.1)	8 (16.0)
				
Mean [Standard deviation]	28.05 [5.13]^a^	28.31 [5.57]^b^	25.34 [4.34]^c^	29.23 [5.77]^d^
				
Ethnicity				
Hausa	5 (3.4)	1 (0.7)	0 (0.0)	1 (2.0)
Ibo	15 (10.1)	7 (5.1)	2 (6.3)	2 (4.0)
Yoruba	126 (85.1)	128 (93.4)	30 (93.8)	47 (94.0)
Other	2 (1.4)	1 (0.7)	-	-
				
Marital status				
Not Married	4 (2.7)	2 (1.5)	1 (3.1)	0 (0.0)
Married	144 (97.3)	135 (98.5)	31 (96.9)	50 (100.0)
				
Parity				
Primiparous	51 (34.5)	57 (41.6)	15 (46.9)	21 (42.0)
Multiparous	97 (65.5)	80 (58.4)	17 (53.1)	29 (58.0)
				
Religion				
Christianity	49 (33.1)	32 (23.4)	10 (31.3)	8 (16.0)
Islam	99 (66.9)	105 (76.6)	22 (68.8)	42 (84.0)
				
Religion of spouse				
Christianity	48 (32.4)	36 (26.3)	9 (28.1)	8 (16.0)
Islam	100 (67.6)	101 (73.7)	23 (71.9)	42 (84.0)
				
Education				
None	3 (2.0)	5 (3.6)	1 (3.1)	2 (4.0)
Primary	22 (14.9)	25 (18.2)	4 (12.5)	11 (22.0)
Secondary	103 (69.6)	95 (69.3)	24 (75.0)	32 (64.0)
Tertiary	20 (13.5)	12 (8.8)	3 (9.4)	5 (10.0)
				
Education of spouse				
None	0 (0.0)	4 (2.9)	1 (3.1)	2 (4.0)
Primary	5 (3.4)	5 (3.6)	0 (0.0)	1 (2.0)
Secondary	98 (66.2)	99 (72.3)	24 (75.0)	37 (74.0)
Tertiary	45 (30.4)	29 (21.2)	7 (21.9)	10 (20.0)
				
Occupation				
None	24 (16.2)	15 (10.9)	5 (10.0)	5 (15.6)
Small trade	88 (59.5)	81 (59.1)	34 (68.0)	21 (65.6)
Casual job	4 (2.7)	8 (5.8)	2 (4.0)	2 (6.3)
Full-time job	32 (21.6)	33 (24.1)	9 (18.0)	4 (12.5)
				
Occupation of spouse				
None	7 (4.7)	1 (1.0)	1 (3.2)	0 (0.0)
Small trade	33 (22.3)	18 (17.5)	7 (22.6)	3 (17.6)
Casual job	3 (2.0)	7 (6.8)	1 (3.2)	3 (17.6)
Full-time job	105 (70.9)	77 (74.8)	22 (71.0)	11 (64.7)

(a) versus (b): t = 0.41, df = 281, p = 0.683; (c) versus (d): t = 3.25, df = 78, p = 0.002

**Table 3:** Profile of infants who completed or did not complete hearing screening programme

**Profile**	**Two-stage screening**		**Diagnosis**	
	
	**Did not complete (%)** n = 148	**Completed (%)** n = 137	**Did not complete (%)** n = 32	**Completed (%)** n = 50
Chronological age (Days)				
1–30	104 (70.3)	98 (71.5)	22 (68.8)	11 (21.6)
31 – 60	24 (16.2)	25 (18.2)	7 (21.9)	159 (20.0)
61 – 90	20 (13.5)	14 (10.2)	3 (9.4)	134 (31.3)
				
Mean [Standard deviation]	24.58 [25.49]	24.26 [21.71]	25.91 [23.16]	28.88 [24.97]
				
Gestational Age (Weeks)				
<37	6 (4.1)	3 (2.2)	0 (0.0)	2 (4.0)
≥37	142 (95.9)	134 (97.8)	32 (100)	48 (96.0)
				
Mean [Standard deviation]	38.08 [1.23]	38.15 [1.27]	38.31 [0.74]	37.86 [1.54]
				
Sex				
Female	67 (45.3)	65 (47.4)	15 (46.9)	20 (40.0)
Male	81 (54.7)	72 (52.6)	17 (53.1)	30 (60.0)
				
Place of birth/delivery‡				
Outside Hospital	82 (55.4)	92 (67.2)	21 (65.6)	35 (70.0)
Hospital	66 (44.6)	45 (32.8)	11 (34.4)	15 (30.0)
				
Hospital admission in the first 28 days of life				
Yes	13 (8.8)	17 (12.4)	5 (15.6)	6 (12.0)
No	135 (91.2)	120 (87.6)	27 (84.4)	44 (88.0)

‡ In the regression model for predicting completion of two-stage screening only place of delivery was significant at alpha level p < 0.10 after adjustment for maternal age, religion and occupation (odds ratio: 1.62, 95% confidence interval: 0.98 – 2.70; p = 0.062). There was no prediction model for completion of diagnostic test as no factor was significant in the univariable analyses.

Only a few mothers who did not complete either the two-stage screening or diagnostic evaluation had little or no formal education. There were no significant differences among mothers who did not complete the second-stage screening (mean: 28.31 ± 5.57 years) and those who completed (mean: 28.05 ± 5.13 years) across virtually all the factors except that those who completed second-stage screening were significantly more likely to have delivered outside hospitals compared to those who did not complete screening (OR: 1.65, 95% CI: 1.02 – 2.66, p = 0.042) in the univariable analysis. However, the strength of this association was reduced (OR: 1.62, 95% CI: 0.98 – 2.70; p = 0.062) after adjusting for maternal age, religion and occupation (Tables 2 &3). There was no evidence of significant interactions of variables in the model and of poor model calibration (Hosmer-Lemeshow test: p = 0.964). The model's discriminatory ability was low (c-statistic = 0.604) and only a small proportion of variation in screening completion was explained by place of delivery (Nagelkerke *R*^2 ^= 0.044).

In contrast, there were no significant differences between those who did not complete diagnostic evaluation and those who completed as no factor was significantly associated with completion of diagnostic evaluation in the univariable analyses. However, mothers who did not complete diagnostic evaluation (mean: 25.34 ± 4.34 years) were significantly younger than those who completed (t = 3.25, df = 78, p = 0.002) as shown in Table 2. The proportion of those who did not complete diagnostic evaluation that delivered in hospitals was not significantly different from those who completed the evaluation.

## Discussion

Although the vast majority of mothers from an inner-city population will participate in an infant hearing screening programme regardless of where they delivered their babies this study has shown that a high proportion may not complete the stages subsequent to the initial screening. This study also suggests that the reasons for this practice are less likely to be associated with educational attainment as vast majority of mothers had a minimum of secondary education. In fact, at least 83% of mothers who did not complete the second-stage screening or diagnostic evaluation had a minimum of secondary education. It was also unlikely that ability to pay for services was a major barrier as all the services under this programme were offered free of charge and none of the perinatal profile of the infants examined was associated with non-compliance.

Mothers in an inner-city environment well served by several public and private hospitals usually choose the care of traditional birth attendants primarily for the mother's safety (due to prevailing superstitious beliefs on the risk of childbirth) rather than the well-being of the newborn. After safe delivery, such mothers may show greater enthusiasm in seeking modern services usually offered to babies delivered in hospitals to ensure that their babies are not unduly disadvantaged. This was evidenced by the higher uptake for BCG immunisation among these infants shortly after birth as about 86% were presented for immunisation within the first month of life (data not shown). This may explain why such mothers were found to have better compliance with follow-up services than those who delivered in hospitals. Reasons why younger mothers were less likely to complete the screening programme protocol which is consistent with the findings of Folsom and colleagues [13] albeit in a developed country also merit further investigation.

Undoubtedly, it would appear that factors other than those examined in this study could have predominantly accounted for these poor return rates or help to explain the impact of place of delivery and maternal age on the completion of the screening protocol. For instance, while factors such as unfavourable attitudes and superstitious beliefs towards childhood deafness and other disabilities in developing countries or the stigma often associated with deafness were not specifically explored, it was not unlikely that they would have contributed in some ways to poor return rates among mothers. For example, among the predominant ethnic group in this study population, having a child with hearing loss is perceived as a curse, a spiritual attack or divine punishment from a deity which is a source of stigma and shame for the affected family [19,20]. In this culture, being childless is preferred to having a child with abnormality. This attitude, which is associated with infanticide, is also not uncommon in other developing countries [21,22]. The impact is often minimal during the first-stage screening because all infants regardless of their hearing status are screened and the vast majority usually pass if the tests are well conducted. Despite assurances given to mothers when the screening results were communicated to them by the screening staff and the uncertainty of the diagnostic outcome at this stage, a referral at any stage for some mothers may have provoked some anxiety about the possibility of a hearing loss and the associated consequences in an apparently normal child which they were afraid of or reluctant to face at such an early age. Most parents promised to return but did not. Efforts to address this problem necessarily should extend beyond individual counselling of parents to include community-based health promotion towards a cultural re-orientation. Such initiatives may be complemented by active media engagement that emphasises the benefits of early detection and intervention through real life examples as successfully demonstrated with similar community-oriented programmes [17,23].

The perception of hearing loss as non-life-threatening in an environment characterised by an overwhelming attention to child survival and little interest on quality of life issues for the survivors was also a potential barrier to high return rates. The notion that if a health condition 'doesn't kill, it doesn't hurt' or the health providers' attitude of if it 'doesn't kill it, can wait' is typical in this population. So, mothers who shared their experience in the screening programme with other health professionals who were not familiar with the programme were unlikely to get any incentive or strong motivation to return for re-screening. In fact, some health professionals are still not aware or convinced that infants can be reliably tested until they are older and at the time when probably speech delay is evident [24,25]. A major public health campaign sponsored or backed by government along with initiatives from the academic community particularly from within the developing world will be valuable in addressing this issue [5].

This programme relied substantially on voluntary compliance of mothers to return for follow-up appointments with minimal prompting from the screening staff which may have also affected the return rates adversely. The only reminder for majority of parents was an appointment slip. A good number of mothers were unreachable by phone but the vast majority who returned were contacted through their mobile phones. Experiences from successful infant hearing programmes in more advanced countries have shown that effective tracking and follow-up of mothers was critical to achieving good return rates [11]. This often entailed setting up centralised database for all child health services to facilitate easy identification of babies with pending appointments for any intervention including hearing evaluation. Within the context of a developing country with poorer infrastructure such a tracking system may be impracticable in the short run. However, improved return rates can be achieved by investing in a dedicated team for follow-up rather than entrusting this function to the screening team as was the case in this study.

The limited attempts made to follow-up the defaulting mothers revealed additional challenges to be addressed for effective tracking under the programme. For example, many infants were lost to follow-up due to untraceable or fictitious contact addresses. Addresses given by some mothers were bus stops, business centres, markets and public buildings. Another factor was the often overlooked influence of spouses in maternal health-seeking behaviour. Because the first-stage screening took place at the screening site it did not require the involvement of spouses. However, bearing in mind that most (>95%) mothers were married the decision to attend follow-up clinics would necessarily have involved the spouses who unfortunately did not benefit from the educational talks on the importance of early hearing detection offered at ante-natal clinics or screening sites. Culturally, Nigeria like many other developing countries is largely a paternalistic society where spouses usually wield considerable influence on maternal health-seeking behaviour especially when it affects the child. Public health campaigns would therefore need to be extended to spouses on the benefits of infant hearing screening possibly through local electronic media.

Mobile telephone contacts proved useful in tracking some defaulters although incomplete or inaccurate numbers was a common problem. Other problems encountered with tracking by mobile phone were similar to tracking with addresses and included numbers that were disconnected, business centres, family, friends, neighbours or distant relations/unknown persons. Some mothers may have deliberately given fictitious contact details as has been observed with similar programmes even in developed countries [26]. Nonetheless, in majority of cases, mobile phone numbers proved quite useful in tracking follow-up appointments. Although in the minority, working mothers also experienced great difficulty in keeping follow-up appointments because they were likely to be constrained to leave their offices during working hours having just resumed work after the statutory 90 days maternity leave, except on account of a serious illness. The problems of living and working in a busy commercial city like Lagos could have also made it difficult for some mothers to keep the necessary appointments for their children without the help of friends or family. It may therefore be worthwhile for instance, to explore the effectiveness of using the several visits mothers make to the health centres in the first year of delivery for other routine immunisations subsequent to the BCG vaccination such as diphtheria-pertussis-tetanus (DPT) and polio as platforms for further parental education and counselling to remind parents of the importance of early detection and consequences of late detection of PCEHL. These clinics can also be used to set up a tracking system including the use of reminder stickers in a way that does not compromise uptake for immunisation. An on-going pilot study is already seeking to reduce the number of visits following the initial referral by providing the second-stage screening before the mothers leave for home in a manner that will not be detrimental to the primary intervention programme thus limiting return visits only for diagnostic services which presently cannot be offered at the immunisation clinics. While delaying repeat TEOAE tests often reduces the incidence of false-positives, given the average age (17.1 days) of infants attending these clinics, this risk is likely to be lower than in a typical hospital-based programme [5].

An area of concern is the likely impact of introducing user fees for infant hearing screening in Nigeria and many other developing countries in line with the current pattern of healthcare financing where patients are required to bear the (full or partial) cost of virtually all healthcare services. Introduction of user fees may become necessary for the rapid and systematic scaling up of early hearing detection and intervention services nationwide because of limited public funding. However, studies have demonstrated substantial reduction in uptake of services and return rates when such user fees are introduced. For instance, a study in a private hospital in South Africa reported a subtantial drop in uptake of hearing screening from 75% when screening was subsidised to 20% when the subsidy was withdrawn [27]. Given the public health significance of early hearing detection and intervention it is necessary for government to consider ways of mitigating their adverse effects based on the experiences with other health interventions. In Nigeria for example, one option is to make all services under this programme eligible for coverage under the country's newly introduced National Health Insurance Scheme. However, patients would still be required to bear the transportation costs of returning for follow-up services.

Overall, the findings in this study are not only applicable to other developing countries but should also provide useful insights for effective delivery of infant hearing screening services to immigrant populations from this region residing in developed countries. However, it is essential to set the results of this study against the backdrop of the fact that this community-based programme was the first newborn or infant screening service ever to be provided in this population unlike the practice in many developed countries where universal newborn hearing screening are preceded by other newborn screening programmes. In this circumstance, it would have been useful to have tested the patient's understanding of the information presented prior to screening with a structured presentation as this has been associated with some success [28]. Given the limited performance of our prediction model it may also be helpful to examine other variables such as maternal obstetric history, income status and family size which were not included in this study. Future studies addressing these issues as well as the impact of culture and beliefs should therefore be considered. For example, a study using an adapted version of the Health Belief Model would be valuable to establish the possible role of individual and community beliefs on deafness across the domains of perceived susceptibility, severity, benefits and barriers [15,29].

## Conclusion

As community-based infant hearing screening programmes emerge in developing countries initial maternal enthusiasm, convenient delivery and the absence of user fees are likely to result in high participation rates. However, a variety of factors besides maternal age and place of delivery are likely to militate against the completion of multi-stage screening protocols by infants who require further assessment. Further research on the likely role of the prevailing superstitious beliefs related to childhood disabilities and the extent to which these factors can be minimised through pre-screening parental education and counselling would be helpful. In the interim, it may be worthwhile to reduce return visits by mothers to the barest minimum by concluding the required screening stages on the first visit to the clinics. Other routine childhood immunisations in the first year of life may be considered also as vehicle for reminding mothers of infants who require follow-up services and for fostering effective tracking system.

## Abbreviations

**AABR**: Automated auditory brainstem response; **ABR**: Auditory brainstem response; **BCG**: bacille de Calmette-Guérin; **CI**: confidence interval; **DPT**: diphtheria-pertussis-tetanus; **JCIH**: Joint Committee on Infant Hearing; **OR**: Odds ratio; **PCEHL**: Permanent congenital and early-onset hearing loss; **TEOAE**: Transient evoked otoacoustic emissions; **UNHS**: Universal newborn hearing screening

## Competing interests

The authors declare that they have no competing interests.

## Authors' contributions

BOO designed the study as part of a wider doctoral research project. OA was involved in follow-up, parental counselling at various stages and data collection. BOO drafted the manuscript and was reviewed and approved by OA. All authors read and approved the final manuscript. BOO is guarantor of the paper.

## Pre-publication history

The pre-publication history for this paper can be accessed here:


